# *Ganoderma Lucidum* from Red Mushroom Attenuates Formaldehyde-Induced Liver Damage in Experimental Male Rat Model

**DOI:** 10.3390/biology9100313

**Published:** 2020-09-27

**Authors:** Babatunde Oluwafemi Adetuyi, Tolulope Olamide Okeowo, Oluwatosin Adefunke Adetuyi, Oluwaseun Abraham Adebisi, Olubanke Olujoke Ogunlana, Oyeyemi Janet Oretade, Najat Marraiki, Amany Magdy Beshbishy, Nermeen N. Welson, Gaber El-Saber Batiha

**Affiliations:** 1 Department of Natural Sciences, Faculty of Pure and Applied Sciences, Precious Cornerstone University, Ibadan P.M.B 234, Nigeria; 2 Department of Biochemistry, Adekunle Ajasin University, Ondo State P.M.B 001, Nigeria; olamidetoluwalope50@gmail.com; 3 Department of Biochemistry, Osun State University, Osogbo P.M.B 4494, Nigeria; adetuyioluwatosin.oa@gmail.com (O.A.A.); adebisiseun15@gmail.com (O.A.A.); 4 Department of Biochemistry, Covenant University, Ota, Ogun State P.M.B 1023, Nigeria; banke.ogunlana@covenantuniversity.edu.ng; 5 Department of Physiology, Osun State University, Osogbo P.M.B 4494, Nigeria; oretadeoyeyemi@gmail.com; 6 Department of Botany and Microbiology, College of Science, King Saud University, Riyadh 11451, Saudi Arabia; najat@ksu.edu.sa; 7 National Research Center for Protozoan Diseases, Obihiro University of Agriculture and Veterinary Medicine, Nishi 2-13, Inada-cho, Obihiro 080-8555, Japan; amanimagdi2008@gmail.com; 8 Forensic Medicine and Clinical Toxicology Department, Faculty of Medicine, Beni-Suef University, Beni-Suef 62511, Egypt; nermeennemr@yahoo.com; 9 Department of Pharmacology and Therapeutics, Faculty of Veterinary Medicines, Damanhour University, Damanhour 22511, Egypt

**Keywords:** *Ganoderma lucidum*, formaldehyde, liver, oxidative stress, inflammation

## Abstract

**Simple Summary:**

Formaldehyde exposure is common due to inhalation and its presence in some food additives. Upon exposure to formaldehyde via any route, it is majorly metabolized by the liver. However, this metabolism impacts negatively on the liver, and in certain concentrations can result in liver damage referred to as hepatotoxicity. This toxicity is evident by a decrease in antioxidant markers as well as an increase in liver function enzymes, inflammatory markers as well as lipid profile in Wistar rats as shown by this study. To combat the deleterious effect of formaldehyde exposure, this study has shown that *Ganoderma lucidum* from red mushroom presents an excellent natural resource by ameliorating the aforementioned liver toxicity markers. This study should serve as a deterrent for those in the practice of using formaldehyde as food additives. Environment inspectors and governments should ensure that formaldehyde is kept below its toxicity threshold in work environments. However, in cases where hepatotoxicity has ensued or is suspected, *Ganoderma lucidum* could serve as a way to combat this toxicity but should be used under appropriate medical expert supervision.

**Abstract:**

The majority of liver-related illnesses are caused by occupational and domestic exposure to toxic chemicals like formaldehyde (FA), which is widely common in Africa and the world at large. Hence, measures should be taken to protect humans from its hazardous effects. This study, therefore, examines the protective potential of *Ganoderma lucidum* (100 mg/kg body weight) on formaldehyde-induced (40%) liver oxido-inflammation in male rats. Male Wistar rats, 150–200 g, were allotted into four groups of 10 animals as follows: Group 1 was orally treated with 1 mg/mL distilled water, Group 2 was exposed to a 40% formaldehyde vapor environment for 30 min per day, Group 3 was orally treated with 100 mg/kg ethanol extract of *Ganoderma lucidum*, and Group 4 was co-administered formaldehyde and 100 mg/kg ethanol extract of *Ganoderma lucidum*. Rats were then sacrificed 24 h after administering the last dose of treatment, and the livers were excised. *Ganoderma lucidum* significantly reversed the formaldehyde-mediated reduction in body and organ weight. *Ganoderma lucidum* administration significantly prevented oxido-inflammation by reducing the levels of hydrogen peroxide and malondialdehyde and increasing the activity of antioxidant enzymes and glutathione contents, as well as the normal level of nitrite and myeloperoxidase production in FA-treated rats. Additionally, *Ganoderma lucidum* reversed a large decline in proinflammatory markers in formaldehyde. Furthermore, *Ganoderma lucidum* restores formaldehyde-induced histological alterations in the liver. Collectively, our results provide valuable information on the protective potential of *Ganoderma lucidum* in protecting formaldehyde-induced liver oxido-inflammation in male rats.

## 1. Introduction

Occupational and household formaldehyde is a common hydrophilic compound that is immediately retained through the lungs and, to a much lower extent, the skin. Health effects related to its exposure are pronounced when the body at sites like the eye, nose, skin, and throat has direct contact with the compound [1,2]. Researchers have deduced the relationship between the health effects and range of exposures, with some individuals becoming symptomatic at low levels of exposure. A few people may have gentle uneasiness while others have moderate or no inconvenience at comparative exposures. Mean level exposures are at their most elevated in the clinical dissection room or morgue [3]. Formaldehyde (FA) is a colorless, combustible and extremely reactive chemical at standard pressure and temperature [4]. It is broken down in the air and highly stable in liquid [5]. It rapidly diffuses in any tissues, e.g., the liver, through the oral or intraperitoneal route since it collaborates with various cell components [6]. Formalin was first used as a fixative and treating liquid; however, these days it is used in every field of daily life. The most appalling use of formalin is as food additive [7] and that’s why there is a drastic increase in human exposure to formalin intoxication. After intake, FA is readily absorbed from the gastrointestinal tract. In the liver, FA is largely metabolized to methanol and formate by aldehyde dehydrogenase 1 or mitochondrial aldehyde dehydrogenase 2, respectively. However in high concentrations of FA, toxicity arises in the hepatocytes [8]. 

Research conducted on FA exposure to animals shows hepatotoxicity and abnormal histological alterations in the gastrointestinal tract [9]. A low dose of FA has been shown to be mutagenic and carcinogenic and can manifest in a wide range of toxicities in different organs [10]. Gastrointestinal cancer can also be caused by drinking water containing a high concentration of FA [11]. In growing countries like India and Nigeria, the haphazard use of FA in lots of food items and drinking water has exposed a large percentage of citizens to a huge health hazards such as liver damage [12]. The liver is a large, composite organ that performs very important tasks in sugar, fat and protein digestion. It functions in the detoxification of metabolic wastes like ammonia. Together with the spleen, it is associated with the obliteration of the remnants of the erythrocyte and the re-use of its constituents. Bile synthesis and secretion are also present in the liver, lipoproteins and plasma proteins synthesis, as well as coagulating factors. It maintains a steady level of blood glucose via glycogenesis, glycogenolysis and gluconeogenesis. The liver also plays a significant role in the elimination and detoxification of drugs. Therefore, xenobiotics (for example, liquor and numerous drugs), malnutrition, infection, and anemia, can induce liver damage [13]. Hepatic damage is a common disease that mostly occurs as a result of oxidative stress and involves progressive growth from steatosis to hepatocellular carcinoma [14].

Over 2 millennia, most Chinese medicines have made use of fungi for the management of a range of diseases [15]. Traditional oriental therapies have also benefited greatly from medicinal mushrooms, and fungal metabolites are widely used in the treatment of diseases. [16]. Additionally, mushrooms should not only be considered as food, as research has shown that they contain a lot of biologically active compounds [17]. Mushrooms have numerous compounds with some biological significance. The extensive list incorporates polysaccharides, phenolics, proteins, polysaccharide–protein complexes, lipid components, and terpenoids, alkaloids, little peptides and amino acids, nucleotides and nucleosides [18]. This extensive list refers to an extraordinary combination of organic properties, including cancer prevention agents [19], antitumor [20], antimicrobial [17], immunomodulatory [21], anti-inflammatory [22], antiatherogenic [23] and hypoglycemic activities [24]. *Gandoerma lucidum* (Lingzhi, Reishi), which has been used for quite a long time in Asian nations to improve wellbeing and advance life span, is widely perceived as a means of avoiding and treating many diseases, including malignant growth [25]. As far back as 1986, the lethal dose (LD_50_) has been reported to be 5000 mg/kg [26]. In 2006, reports investigated the beneficial roles of *G. lucidum*. Although the vast majority of persuasive information depends on laboratory and preclinical investigations, *G. lucidum* has gained consideration in non-Asian nations [27].

Investigations with refined *G. lucidum* triterpenes have indicated in vivo results, which can be used for drug development. However, the full use of *G. lucidum* preparation in corresponding and alternative medications is progressively beneficial because the specific component of *G. lucidum* could have synergistic or added substance impacts and could influence molecular signaling pathways and targets, finally prompting the destruction of malignant cells. This study is therefore performed to examine the protective potential of *G. lucidum* in formaldehyde-induced liver damage in experimental rats.

## 2. Materials and Methods

### 2.1. Fungi Material and Extraction

The whole basidiomata of *G. lucidum* was obtained from a village in Edo state. Identification was conducted at the Department of Agricultural Sciences, Joseph Ayo Babalola University. *G. lucidum* was air dried away from the direct sun rays and samples were milled to a total of 413 g of boorish powder. In total, 200 g of the quantity of coarse powder was soaked in 1 L ethanol for 72 h, decanted and concentrated, thus yielding a dark brown extract. The extract was weighed and stored in the refrigerator.

### 2.2. Chemicals

Thiobarbituric acid, 1-chloro-2,4-dinitrobenzene (CDNB), 5′,5′-dithiobis-2-nitrobenzoic acid (DTNB), xynelol orange, reduced glutathione (GSH), epinephrine and hydrogen peroxide (H_2_O_2_) were purchased from Sigma Chemical Co. (St. Louis, MO, USA). Other chemicals and reagents were obtained from Cloud-Clone Inc., Wuhan, China.

### 2.3. Animals Care

Forty sexually matured Wistar strain male rats with the weight range of 150–200 g, were acquired in the animal colony, University of Ibadan, Nigeria. They were housed in a polycarbonate cage in an Assessment and Accreditation of Laboratory Animal care-certified animal facility and adherence to the protocol of the National Institute of Health on the Guide for the Care and Use of Laboratory Animals. Prior to acclimatization for 2 weeks, rats were kept under 12:12-h light:dark cycle and provided with NIH-07 diet and water *ad libitum*.

### 2.4. Experimental Design

Forty sexually matured Wistar strain male rats were divided into four groups of ten rats each and treated for thirty days (2 weeks of acclimatization inclusive) as described thus:

Group 1: were orally treated with 1 mg/mL distilled water.

Group 2: were exposed to 40% FA vapor environment for 30 min daily (the exposure was done by soaking 50 mL of FA in cotton wool and placed in a corner within the animal cage, thus exposing the animal to the vapor for a period of 2 weeks (40% FA at room temperature) [28].

Group 3: were orally treated with 100 mg/kg ethanol extract of *G. lucidum*.

Group 4: were co-administered FA and 100 mg/kg ethanol extract of *G. lucidum* (1/50 of LD_50_). The route of administration of G. lucidum was oral and that of FA was the same as in Group 2.

Rats were then sacrificed 24 h after the last administration via cervical dislocation. Liver samples were excised, weighed, homogenized, and then processed for further experiments.

### 2.5. Determination of Liver Function Parameters

Blood samples were collected after sacrifice and plasma samples were obtained using the standard method. Liver function biomarkers, including alanine aminotransferase (ALT), aspartate aminotransferase (AST), alkaline phosphatase (ALP), triglycerides, total bilirubin, direct bilirubin, albumin and cholesterol were assayed according to the manufacturer’s procedure (Randox Laboratories, Crumlin, UK).

### 2.6. Estimation of Antioxidant and Oxidative Stress Markers

The excised liver samples were homogenized accordingly using a 50 mM Tris–KCl buffer at pH 7.4 consisting of 1.15% KCl, then further centrifuged at 12,000× *g* for 15 min at 4 °C and afterward used for biochemical assays. Estimation of the protein concentration was done according to Bradford [29]. Claiborne [30] and Misra and Fridovich [31] methods were used to determine the activities of Catalase (CAT) and superoxide dismutase (SOD). Glutathione-S-transferase (GST) activity and the GSH level were determined according to Habig et al. [32] and Rotruck et al. [33]. Meanwhile, the level of lipid peroxidation (LPO) was determined according to Jollow et al. [34]. H_2_O_2_ generation was determined according to the standard method of Wolff [35]. All biochemical experiments were analyzed using a SpectraMax plate reader (Molecular Device, San Jose, CA, USA).

### 2.7. Assessment of Inflammatory Biomarkers

Myeloperoxidase (MPO) activity was determined according to the method described by Granell et al. [36], whereas the nitrite level concentration was assessed using an established protocol [37].

### 2.8. Determination of Proinflammatory Cytokines

Tumor Necrosis Factor (TNF-α), IL-1β and IL-6 concentrations in liver homogenates samples were assayed using rat TNF-α, IL-1β and IL-6 Elisa kits, respectively (Cloud-Clone Inc., Wuhan, China). A microplate antibody-coated plate was provided with the kit. All reagents, samples and working standards were prepared using standard procedures as provided by the kit manufacturers.

### 2.9. Histological Examination

Liver samples of rats that were removed were fixed with Bouin’s solution which was subsequently dehydrated in graded concentrations of alcohol. This was further cleared three times using xylene solution and was later embedded in paraffin wax. Microtome was then used to cut 4–5 mm of the paraffin waxed tissue on a slide and it was stained with haematoxylin (H) and eosin (E). The slides were then further viewed using a light microscope (Olympus CH; Olympus, Tokyo, Japan) and were snapped by pathologists.

### 2.10. Ethical Approval

All procedures involving animals performed in the study were performed in accordance with the ethical standards of our institution.

### 2.11. Statistical Analyses

Data were evaluated as mean ± SEM. Levels of statistical significance were analyzed with a one-way analysis of variance (ANOVA) which was further subjected to Bonferroni’s post hoc test using GraphPad Prism 6 software. *p* < 0.05 was considered significant.

## 3. Results

### 3.1. G. lucidum Suppressed FA-Induced Reduction in Body and Organ Weight

Table 1 represents the body weight gain and relative organ weight of control, *G. lucidum* and FA-treated rats. The result shows a significant reduction in the body and organ weight of the liver of rats administered formaldehyde as compared to the control. Additionally, there was a statistically significant increase in the body and organ weight gain of rats exposed to FA and *G. lucidum* when compared to the control.

### 3.2. G. lucidum Inhibits FA-Induced Alteration in Hepatic Function Enzymes

**Figure 1:**
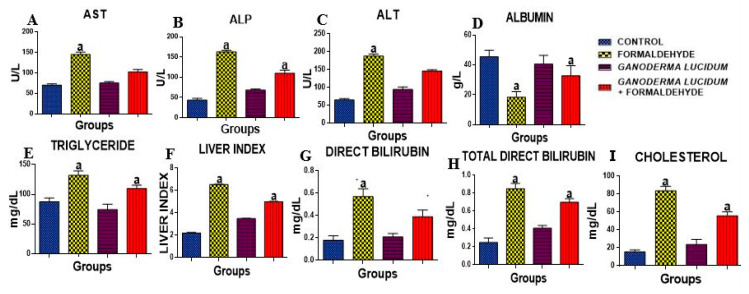
Effect of formaldehyde and *Ganoderma lucidum* on hepatic enzyme markers. Data are presented as mean ± SD, *n* = 7; a: *p* < 0.05 vs. control; (**A**) AST (**B**) ALP (**C**) ALT (**D**) ALB (**E**) TRIG (**F**) LIVER INDEX (**G**) DIRECT BIL (**H**) TOTAL BIL (**I**) CHOL. AST: Aspartate aminotransferase, ALP: Alkaline Phosphatase, ALT: Alanine aminotransferase.

The activities of AST, ALT and ALP were significantly (*p* < 0.05) increased in the liver of rats administered FA as compared to the control. However, *G. lucidum* significantly reduced these levels when compared to the FA alone and control groups (Figure 1A–C). Additionally, the concentration of total cholesterol, direct bilirubin and total direct bilirubin was significantly increased in the liver of rats administered FA as compared to the control (Figure 1G–I). However, *G. lucidum* significantly reduced these levels when compared to the FA alone and control groups. The concentration of ALB was significantly (*p* < 0.05) decreased when compared to the control, but *G. lucidum* reversed this effect. The liver index shows an increase in rats administered FA as compared to the control. However, *G. lucidum* reversed this effect with an increase in the rats co-administered *G. lucidum* and FA (Figure 1F).

### 3.3. G. lucidum Attenuate FA-Induced Oxidative Damage in Rat Liver

**Figure 2:**
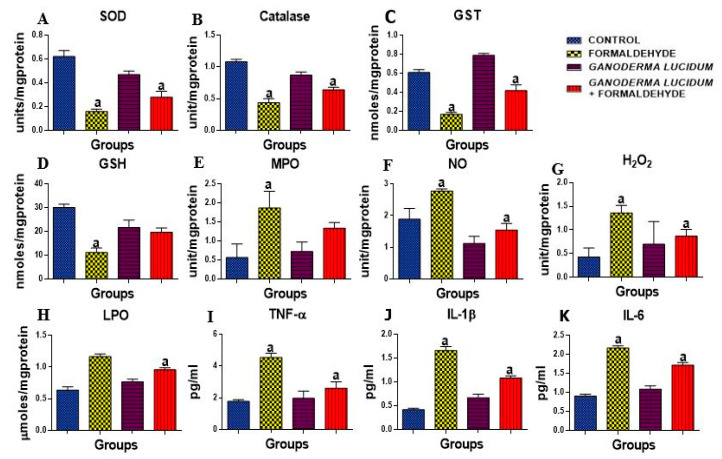
Effect of formaldehyde and *Ganoderma lucidum* on oxido-inflammatory markers. Data are presented as mean ± SD, *n* = 7; a: *p* < 0.05 vs. control, (**A**) SOD: superoxide dismutase, (**B**) CAT: catalase (**C**) GST: Glutathione-s-transferase (**D**) GSH: reduced glutathione, (**E**) MPO: myeloperoxidase, (**F**) NO: Nitric oxide (**G**) H_2_O_2_: Hydrogen peroxide (**H**) LPO: lipid peroxidation (**I**) TNFα: Tumor necrosis factor α. (**J**) IL-1β: Interleukin 1β, (**K**) IL-6: Interleukin 6.

The antioxidant enzymes activities, SOD, CAT, GST and GSH level (Figure 2A–D), and also oxidative stress indices, H_2_O_2_ and MDA, were tested as presented in Figure 2G–H. There was a significant decrease in the liver activities of SOD, CAT, GST and the level of GSH in rats administered FA alone as compared to the control rats. Conversely, the co-administration of *G. lucidum* restored the level and activities of these enzymes. Furthermore, there was a significant increase in the levels of oxidative stress markers (H_2_O_2_ and MDA) in the liver of rats administered FA. However, the rats co-administered with *G. lucidum* revealed a significant decrease in the levels of H_2_O_2_ and MDA in the testes of treated rats as compared to FA alone as shown in Figure 2A,B.

### 3.4. G. lucidum Ameliorate FA-Induced Inflammation in Rat Liver

Nitrite level and the activity of MPO were determined in the liver of rats as shown in Figure 2E,F. An administration of FA alone resulted in a significant elevation of nitrite level and activity of MPO as compared to the control rats. On the contrary, rats co-administered with *G. lucidum* had a significantly decreased nitrite level and MPO activity compared to the livers of FA-administered rats. However, the administration of *G. lucidum* alone did not have an effect on nitrite level and the activity of MPO. Additionally, proinflammatory cytokines (TNF-α, MPO and IL-1β) were significantly (*p* < 0.05) increased in FA-administered rats when compared to the control as shown in Figure 2I–K; however, *G. lucidum* reversed this effect with a significant (*p* < 0.05) decrease in the proinflammatory cytokines as compared to the control.

### 3.5. Histopathological Observations

**Figure 3:**
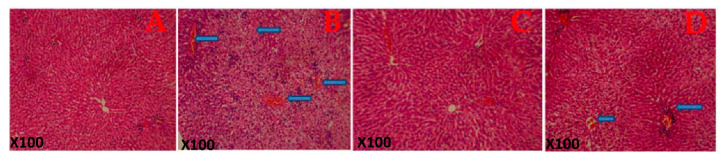
Histological architecture of the liver of the rat in each group. (**A**) Control rat shows the normal architecture of the liver. (**B**) Rats administered Formaldehyde (FA) alone shows a diffuse periportal cellular infiltration with severe congestion indicating hepatic damage. (**C**) Rats administered *Ganoderma lucidum* show a normal architecture of the liver while (**D**) rats co-administered with formaldehyde and *Ganoderma lucidum* shows mild congestion. Magnification: X100.

Figure 3 shows the histological structure of the representative photomicrograph of the liver. The control rats and the *G. lucidum*-treated rats show a normal architecture. However, rats treated with FA alone show a diffuse periportal cellular infiltration with severe congestion indicating hepatic damage. Additionally, histological structures of the liver of rats co-administered with formaldehyde and *G. lucidum* at 100 mg/kg showed relatively normal features.

## 4. Discussion

The use of natural products in the prevention and management of various illnesses has prominently increased in the last few years [38]. The present study established the promising chemopreventive potential of *G. lucidum* in the liver, preventing liver damage caused by FA exposure. The reestablishment of unhealthy liver functions was evident by the remarkable loss of body weight, and a significant reduction in the liver organ weight which can result from shrinkage in the liver as seen in the FA-administered group (Table 1), but this was prevented in the rats treated with *G. lucidum*. The hepatoprotective potential of *G. lucidum* against FA was investigated by determining ALT, AST and ALP. ALT is the important liver damage enzyme that catalyzes transamination reactions. The occurrence of conditions that can cause liver damage such as cancer, injury and hepatitis, will result in higher levels of this enzyme [14]. AST and ALP, the biomarkers of liver damage, are cytosolic and mitochondrial enzymes whose levels are usually increased in cases of chronic illness and necrosis due to loss of hepatocellular integrity. These enzymes are involved in the transfer of α-amino groups from alanine and aspartate to the α-keto group of ketoglutarate to form pyruvate and oxaloacetate, respectively [39]. As shown in figures, there is a significant increase (*p* < 0.05) in the levels of these enzymes in the group administered FA when compared to the control. However, treatment with 100 mg/kg *G. lucidum* significantly reduced the elevated levels, showing that *G. lucidum* exhibits a protective role against FA-induced liver damage in rats. This study is related to that of Lakshmi et al. [40] that showed the effects of *Ganoderma lucidium* on hepatic damage induced by benzo(a) pyrene. The elevated liver function enzymes were significantly reduced by *Gandoerma lucidum* administration.

Albumin is a measure of the synthetic function of the liver. A significant decrease in the albumin level in the FA-administered group could be traced to the reduction in protein synthesis that is an effect of FA. The carbonyl atom of FA reacts with the amino groups (nucleophilic sites) on the cell membranes forming hydroxymethyl amino acid derivatives [41]. However, treatment with 100 mg/kg *G. Lucidum* significantly increases the level of albumin. Cholesterol oxidation causes enzymatic increases in bile acids and contributes to hepatic cholesterol accumulation and hepatocellular injury. This is further explained by its significant increase in the FA-administered group as compared to the control. However, *G. Lucidum* treatment reduced the elevated cholesterol levels significantly when compared to the control. Total direct bilirubin is also an indicator of the destruction of erythrocytes and the proper functioning of the liver, gallbladder and bile ducts, and is a potential marker for liver damage. Triglycerides were also increased in the FA-administered group as compared to the control. However, *G. Lucidum* reduced the elevated cholesterol levels significantly when compared to the control. The liver index, the indicator of hepatic manifestation of metabolic disorders, was upregulated in the group administered FA, thus showing an impairment of the liver. However, upon administration of 100 mg/kg *G. lucidum* to the group induced with 40% FA, there was a significant downregulation in the increased liver index.

When the body metabolism is impaired, an increase in the production of toxic molecules such as free radicals and antioxidants, known as free radical scavengers, are needed to reduce or neutralize the free radical formation [42]. Our results show that FA has a direct effect on the hepatocytes and also an indirect effect through the circulatory and immune systems [43]. The hepatic destruction caused by FA causes oxidative stress and produces reactive oxygen species (ROS), as shown in the significant increase in H_2_O_2_ and LPO, which are known to be oxidative stress markers, and also a decrease in GSH, GST, catalase and SOD, which are antioxidant markers. These observations are accordance to Payani et al. [44] who reported that FA exposure significantly reduced the levels of enzymatic and non-enzymatic antioxidants. However, *G. lucidum* significantly increases the activities and levels of these antioxidant markers. These results indicated that animals treated with *G. Lucidum* cause a significant increase in the levels of antioxidant enzymes. These results are in line with the reports of other researchers that allude to the fact that *Gandoderma lucidium* has antioxidant activities both in vivo and in vitro [45,46,47]. These results indicated the hepatoprotective efficacy of *G. Lucidum.* Myeloperoxidase is one of the most important molecules released after the recruitment and activation of phagocytes and it is involved in the production of oxidative stress. Additionally, proinflammatory cytokines activate iNOS during liver injury to abnormally producing NO that contributes immensely to the pathogenesis and evolution of liver damage. The present study shows a distinct increase in the activity and level of MPO and NO in FA-administered rats’ livers as compared to the control. However, the reduced level of MPO and NO following *G. lucidum* treatment shows its potential to prevent inflammation in the liver of rats [48,49].

TNF, IL-1β and IL-6 play a major role in the pathogenesis of liver damage. TNFs are majorly a group of proinflammatory cytokines known to perform a crucial role in the instigation of liver damage with evidence that oxidative stress might act in conjunction with endotoxins to augment TNF production [50]. Interleukin 1β and 6 are potential biomarkers of acute or chronic liver toxicity. TNF, IL-1β and IL-6 are proinflammatory cytokines that are released into the bloodstream from the liver during hepatic toxic injury. Thus, biological agents suppressing these cytokines are known to have demonstrated huge therapeutic potential. As shown in our results, there was a significant upregulation in the levels of these cytokines in rat livers when administered FA. The significant downregulation of the levels of the cytokines was demonstrated in the group treated with 100 mg/kg *G. Lucidum*. This further indicates the hepatoprotective efficacy of *G. Lucidum.* The above result corroborates with the histopathological finding as shown in Figure 3 as rats administered FA show a diffuse periportal cellular infiltration with severe congestion indicating hepatic damage; however, *G. lucidum* was able to reverse this effect [51].

## 5. Conclusions

The results from this study demonstrated that exposure to FA led to a significant decline in antioxidant markers [52], with a concomitant increase in liver transaminases, lipid profile as well as inflammatory markers [53]. Additionally, *G. lucidum* possesses protective roles as it has the ability to restore antioxidant, lipid profile and anti-inflammatory statuses. Hence, *G. lucidum* may be a probable drug candidate to target liver damage [54,55].

## Figures and Tables

**Table 1:** Effect of *Ganoderma lucidum* and formaldehyde on average body weight and relative organ weight in rat.

	Control (g)	Formaldehyde (g)	*Ganoderma lucidum*	*Ganoderma lucidum* + Formaldehyde (g)
Average Body weight	42.35 ± 4.52	18.75 ± 5.45 ^a^	36.74 ± 6.32	25.45 ± 4.46
Relative organ weight	7.32 ± 0.47	4.76 ± 0.78 ^a^	8.76 ± 0.95	6.75 ± 0.52 ^a^

a—significantly different from control.
